# Detecting *Mycobacterium tuberculosis* Infection in Children Migrating to Australia

**DOI:** 10.3201/eid2809.212426

**Published:** 2022-09

**Authors:** Ingrid Laemmle-Ruff, Stephen M. Graham, Bridget Williams, Danielle Horyniak, Suman S. Majumdar, Georgia A. Paxton, Lila V. Soares Caplice, Margaret E. Hellard, James M. Trauer

**Affiliations:** Burnet Institute, Melbourne, Victoria, Australia (I. Laemmle-Ruff, S.M. Graham, B. Williams, D. Horyniak, S.S. Majumdar, M.E. Hellard, J.M. Trauer);; Royal Children’s Hospital, Melbourne (I. Laemmle-Ruff, S.M. Graham, G.A. Paxton);; University of Melbourne, Melbourne (S.M. Graham, S.S. Majumdar, G.A. Paxton, M.E. Hellard, J.M. Trauer);; Monash University, Melbourne (D. Horyniak, S.S. Majumdar, M.E. Hellard, J.M. Trauer);; The Alfred Hospital, Melbourne (S.S. Majumdar, M.E. Hellard, J.M. Trauer);; Department of Home Affairs, Australian Government, Canberra, Australian Capital Territory, Australia (L.V.S. Caplice)

**Keywords:** tuberculosis and other mycobacteria, latent tuberculosis infection, children, screening, migration, bacteria, Australia

## Abstract

In 2015, Australia updated premigration screening for tuberculosis (TB) disease in children 2–10 years of age to include testing for infection with *Mycobacterium tuberculosis* and enable detection of latent TB infection (LTBI). We analyzed TB screening results in children <15 years of age during November 2015–June 2017. We found 45,060 child applicants were tested with interferon-gamma release assay (IGRA) (57.7% of tests) or tuberculin skin test (TST) (42.3% of tests). A total of 21 cases of TB were diagnosed: 4 without IGRA or TST, 10 with positive IGRA or TST, and 7 with negative results. LTBI was detected in 3.3% (1,473/44,709) of children, for 30 applicants screened per LTBI case detected. LTBI-associated factors included increasing age, TB contact, origin from a higher TB prevalence region, and testing by TST. Detection of TB and LTBI benefit children, but the updated screening program’s effect on TB in Australia is likely to be limited.

Tuberculosis (TB) is a leading contributor to the burden of infectious disease worldwide, causing >1.2 million deaths in 2019 (*1*,*2*). Latent tuberculosis infection (LTBI), defined as infection with *Mycobacterium tuberculosis* without clinical or radiologic evidence of disease, can be diagnosed with interferon-gamma release assay (IGRA) or tuberculin skin test (TST). Estimates suggest one quarter of the global population have LTBI, including 97 million children (<15 years of age) (*3*). Young children (<5 years of age) are at particular risk for TB if infected and have a higher risk for disseminated or severe disease associated with severe illness and death. TB preventive treatment can reduce progression to disease by >90% in children with infection, but effectiveness is greatest if preventive treatment is initiated in the months immediately after infection, emphasizing the importance of early detection and treatment (*4*,*5*). Preventive treatment for children usually involves 6 months of isoniazid monotherapy or, less commonly in Australia, 3 months of rifampin/isoniazid combination therapy (*4*).

Treating LTBI is increasingly recognized as a crucial component of global TB elimination efforts (*6*). Reactivation of overseas-acquired LTBI in adult migrants is the main source of TB in low-burden countries such as Australia (*7*,*8*). Decreasing LTBI in migrants is a key means to further reduce TB incidence (*9*,*10*). LTBI is not notifiable in Australia, and local prevalence estimates vary (*11*). Recent modeling estimates that 17.1% of all overseas-born residents of Australia, 2.1% of overseas-born children <15 years of age, and 0.1% of Australia-born children <15 years of age have LTBI (*12*).

Clinical assessment and chest radiography have long been part of premigration TB screening in Australia and other high-resource settings (*13*–*15*). However, the systematic inclusion of IGRA and TB preventive treatment in premigration health assessments is uncommon internationally and relatively novel in children (*16*,*17*). After the United States and Norway, Australia introduced TB screening using IGRA and preventive treatment for children 2–10 years of age on November 20, 2015 (*13*,*18*,*19*). The intention was “to strengthen screening for active TB to improve detection of this disease,” which would “also identify children with LTBI” (*20*). Previously, premigration screening of children <11 years of age had been limited to medical history and examination and included chest radiography only in those with TB contact or where TB was suspected. More broadly, the updated screening program was “designed to improve public health protections in Australia but also contribute to global efforts to eliminate TB” (*19*), reflecting an emerging recognition of the public health potential of premigration health screening (*21*).

In this cross-sectional study, we aimed to assess the first 20 months of Australia’s updated premigration TB screening for children. In particular, we sought to assess the scope of implementation, yield in detecting TB and LTBI, and impacts on follow-up requirements after migration. We present numbers and proportions of children screened, screening results (TB and LTBI), factors associated with LTBI, further investigation, and requirements for linkage to care in Australia.

## Methods

The following sections summarize Australia’s premigration health screening program and the dataset. Further detail is available elsewhere (*22*).

### Screening Program

Migration legislation in Australia requires all permanent, provisional, and humanitarian visa applicants, as well as some temporary visa applicants, to undergo an Immigration Medical Examination (IME) and meet a health requirement before being granted a visa (*23*,*24*). Premigration health screening is intended to protect the Australia community from public health threats, control public expenditure on healthcare and services, and safeguard the access of Australia citizens and permanent residents to healthcare and services in short supply (*20*,*24*). Applicants cannot be granted a visa if they have TB, but the health requirement can be met and a visa granted once TB treatment is complete and the person is free of TB. An IME involves a medical history, examination by a physician, and criteria-based investigations (*20*,*22*,*23*), including criteria for performing IGRA or TB preventive treatment (Table 1). Applicants applying offshore can be assigned a health undertaking, which is an agreement requiring follow-up care in Australia. TB health undertakings (TBHUs) are allocated to applicants with risk factors for TB, including previously treated TB, abnormal chest radiography, and positive IGRA or TST results, and reflect a requirement for linkage to care in Australia in our analysis. Onshore applicants (with their IME conducted in Australia) are referred for care if required without a TBHU.

**Table 1:** Australian premigration TB screening criteria within the Immigration Medical Examination*

All children completing an IME have a medical history and physical examination
Either IGRA or TST is required for:
Children 2–10 years of age who are:
Applying for a humanitarian visa
Applying for a permanent visa and from a setting placing them at higher risk for TB†
Asylum seekers within Australia
Applicants declaring close contact with TB, with signs or symptoms of TB, or who are immunocompromised (any age or migration stream)
Applicants with positive IGRA or TST results are required to have:
Posteroanterior chest radiograph (and lateral in children <11 years of age)
If abnormalities on chest radiograph, or other indication for further investigation:
Sputum testing and specialist review
Exemptions from IGRA and TST screening:
Written evidence of prior bacteriologically confirmed TB (i.e., positive smear or culture from sputum or other specimen) or a previously positive TST (>10 mm) or IGRA

*Chest radiograph screening for TB is required for all migrants >11 years applying for permanent or humanitarian visas and for temporary visas if from high-risk TB countries and staying for >6 months. TB, tuberculosis disease; IME, Immigration Medical Examination; IGRA, interferon-gamma release assay; TST, tuberculin skin test. †Prevalence >40 per 100,000 cases of TB based on 2013 World Health Organization estimates.

### Study Population

The source study population was all permanent and humanitarian visa applicants to Australia and temporary applicants intending to stay for >6 months who completed an IME (onshore or offshore) and met the health requirement or were granted a waiver during July 1, 2014–June 30, 2017. Our analysis is restricted to child applicants (<15 years of age) who completed an IME during November 2015–June 2017, reflecting when new screening commenced. Age was available for analysis in 5-year brackets (0–4, 5–9, and 10–14 years) for privacy reasons. Thus the analysis includes all children 2–10 years of age but also includes children <2 years and 11–14 years who did not meet age-based screening criteria. Deidentified IME data were provided by the Australian Department of Home Affairs. The Alfred Hospital Ethics Committee provided ethics approval (project 320/17).

### Variables

Variables were applicant demographics, visa stream, medical history and examination findings, investigation type and results, physician-recorded diagnoses, and allocation of TBHUs. We defined TB as a recorded diagnosis of TB by the assessing physician. We defined IGRA or TST positivity as having any IGRA or TST result recorded as positive in the IME (from options of positive, negative, and indeterminate). LTBI was defined as IGRA or TST positivity without a diagnosis of TB or previous history of TB.

Bacillus Calmette-Guérin (BCG) vaccination status was not available. Data were supplied as categorical variables, based on the menu option selected by the physician. Because country of birth was not available for all applicants, a country of origin variable was derived hierarchically, using (in order, if available) country of birth, country of travel document, or country of residence (*22*).

### Statistical Analysis

We described results as absolute numbers and proportions. We used the Fisher exact test to compare proportions. We noted missing and indeterminate IGRA and TST results then excluded them from further analysis.

We used univariate and multivariable-logistic regression to identify demographic and clinical factors associated with LTBI among permanent and humanitarian visa applicants. Temporary applicants were excluded from regression analyses because they were not included in screening criteria unless clinical risk factors were present. We included variables with previous evidence of association with LTBI in the regression. Exploratory forward regression methods did not reveal additional variables that substantially altered results. Specifically, relevant comorbidity variables were examined but not included because of negligible prevalence (e.g., among children screened, 14 had diabetes and <5 had HIV). Applicants who had previously been treated for TB were excluded because we assumed this history would result in a positive IGRA or TST without reflecting LTBI. Screened applicants with missing or inadequate country of origin were also excluded from regression analyses. We also performed a sensitivity analysis restricted to applicants 5–9 years of age (i.e., all within the age range of new screening criteria).

## Results

### Participants

During November 2015–June 2017, a total of 134,759 children <15 years of age completed an IME and met the health requirement (Table 2). Of these, 48.6% were girls, 46.5% were 0–4 years of age, and 53.9% were permanent applicants.

**Table 2:** IGRA and TST testing and positivity in visa applicants <15 years of age migrating to Australia, November 2015–June 2017*

Characteristic	All applicants	Applicants tested by IGRA or TST	Applicants tested, excluding missing/indeterminate results†	IGRA or TST positivity in applicants tested
Total	134,759 (100)	45,060 (33.4)	44,841	1,513 (3.4)
Sex				
F	65,462 (48.6)	22,133 (33.8)	22,028	763 (3.5)
M	69,284 (51.4)	22,925 (33.1)	22,811	750 (3.3)
Age group, y				
<4	62,646 (46.5)	18,503 (29.5)	18,410	505 (2.7)
5–9	40,357 (30.0)	22,615 (56.0)	22,509	796 (3.5)
10–14	31,756 (23.6)	3,942 (12.4)	3,922	212 (5.4)
Visa stream				
Permanent	72,610 (53.9)	30,474 (42.0)	30,349	1,144 (3.8)
Temporary	45,490 (33.8)	4,466 (9.8)	4,430	160 (3.6)
Humanitarian	16,659 (12.36)	10,120 (60.8)	10,062	209 (2.1)
Region of origin				
Oceania‡	10,704 (7.9)	2,512 (23.5)	2,490	32 (1.3)
Northwest Europe	7,339 (5.5)	255 (3.5)	255	7 (2.8)
Southern and Eastern Europe	2,153 (1.6)	419 (19.5)	417	36 (8.6)
North Africa and Middle East	14,316 (10.6)	6,981 (48.8)	6,950	128 (1.8)
Southeast Asia	21,993 (16.3)	7,597 (34.5)	7,562	574 (7.6)
Northeast Asia	19,667 (14.6)	5,788 (29.4)	5,755	134 (2.3)
Southern and Central Asia	37,741 (28.0)	15,046 (39.9)	15,009	435 (2.9)
Americas	4,363 (3.2)	503 (11.5)	498	11 (2.2)
Sub-Saharan Africa	8,556 (6.4)	3,557 (41.6)	3,522	97 (2.8)
Missing/Inadequately described	7,927 (5.9)	2,402 (30.0)	2,383	59 (2.5)
Top 5 countries of origin, excluding Australia				
India	25,208	9,860 (39.1)	9,831	317 (3.2)
China	14,013	4,703 (33.6)	4,680	114 (2.4)
Philippines	8,684	3,234 (37.2)	3,222	436 (13.5)
United Kingdom	5,562	170 (3.1)	170	5 (2.9)
Pakistan	5,175	2,472 (47.8)	2,471	24 (1.0)
Reported risk factors				
Reported contact with TB	748	310 (41.4)	306	46 (15)
Previous treatment for TB	402	121 (30.1)	121	31 (25.6)
Test type		No. (%) tests completed§	No. tests, excluding indeterminate	No. (%) positive tests
All		45,345	45,171	1,563 (3.5)
IGRA		26,171 (57.7)	25,997	532 (2.0)
TST		19,174 (42.3)	19,174	1,031 (5.4)

*Values are no. (%) except as indicated. Missing results across all applicants (excluded from corresponding demographic denominators): age not recorded for 7 applicants, none of whom were screened (excluded from entire analysis); gender not recorded for 13 applicants, reported TB contact not recorded for 7,645 applicants, previous treatment for TB not recorded for 13,484 applicants. Humanitarian visa holders, while considered as a separate visa application stream by the Department of Home Affairs, have permanent residency in Australia. IGRA, interferon-gamma release assay; TST, tuberculin skin test; TB, tuberculosis disease. †Applicants who had IGRA or TST but with an indeterminate (IGRA, n = 174) or missing result (n = 45) excluded from column. ‡Oceania region includes 8,358 applicants with country of origin as Australia (8,018 born in Australia). This reflects children born in Australia of families applying for migration. No further information regarding parental country of origin available. §Total of 45,345 IGRAs and TSTs conducted with 330 applicants with >1 test in an application (227 with 2 tests and 3 with 3 tests).

### Screening Completion

IGRA or TST was completed in 45,060 applicants, representing 33.4% of all child applicants (Table 2); 330 children had multiple tests within an application (reasons not available). Of 45,345 tests conducted, IGRA was the most common testing method (57.7%) in all age groups: children 0–4 years of age (11,121/18,573 [59.9%]), 5–9 years of age (12,871/22,792 [56.5%]), and 10–14 years of age (2,179/3,980 [54.7%]). A higher proportion of children 5–9 years of age completed testing than did those in other age groups (p<0.001), consistent with screening criteria targeting the ages of 2–10 years. A higher proportion of humanitarian applicants completed testing compared with other visa streams (p<0.001); for humanitarian applicants 5–9 years of age (all of whom met criteria), IGRA or TST was completed for 5,403/5,734 (94.2%) persons.

The largest number of children in whom IGRA or TST was performed came from Southern and Central Asia, which had 15,046 applicants tested (12,288 permanent, 1,854 temporary, and 904 humanitarian), reflecting the most common region of origin in the largest visa stream (permanent visa applicants). The North Africa and Middle East region had the highest proportion of applicants tested (48.8%), reflecting a predominantly humanitarian applicant population (819 permanent, 57 temporary, and 6,105 humanitarian).

### LTBI Results

Excluding missing and indeterminate results, 1,513/45,060 (3.4%) applicants returned a positive IGRA or TST result (Table 2). In children without TB or a history of treatment for TB, 1,473/44,709 (3.3%) had a positive result, which equates to 3,295 cases of LTBI per 100,000 applicants tested, or 30 applicants screened per LTBI case detected. The proportion of positive results was higher for TST (5.4%) than IGRA (2.0%; p<0.001). Two thirds (1,001/1,513 [66.2%]) of children who tested positive were identified through TST and the remainder were identified through IGRA (512/1,513 [33.8%]; p<0.001). The prevalence of TST positivity increased with age, from 4.6% in children 0–4 years of age to 8.4% in those 10–14 years of age.

The proportion of positive IGRA/TST tests was highest in permanent applicants (3.8%; p<0.001) and applicants 10–14 years of age (5.4%; p<0.001). Applicants from India, China, and the Philippines comprised more than one third of children tested (17,797/45,060 [39.5%]) and more than half of all positive results (867/1,513 [57.3%]).

Factors associated with LTBI (Table 3) included being 10–14 years of age, originating from Southeast Asia or Southern and Eastern Europe, testing by TST, and past close TB contact. Factors negatively associated with LTBI included originating from Oceania, age of 0–4 years, and being a humanitarian applicant. Sensitivity analyses restricted to children 5–9 years of age did not significantly alter these findings.

**Table 3:** Factors associated with LTBI in applicants <15 years of age migrating to Australia, November 2015–June 2017*

Characteristic	Univariate regression analysis			Multivariate regression analysis	
	Odds ratio (95% CI)	p value		Odds ratio (95% CI)	p value
Sex					
M	Referent	0.449		Referent	0.481
F	1.04 (0.93–1.17)			1.04 (0.93–1.17)	
Age group, y					
0–4	0.78 (0.69–0.88)	<0.001		0.84 (0.74–0.95)	0.007
5–9	Referent			Referent	
10–14	1.55 (1.31–1.83)	<0.001		1.40 (1.17–1.65)	<0.001
Region of origin					
Northeast Asia	Referent			Referent	
Oceania	0.48 (0.31–0.75)	0.001		0.53 (0.35–0.83)	0.005
Northwest Europe	0.68 (0.21–2.16)	0.515		0.55 (0.17–1.77)	0.318
Southern and Eastern Europe	3.35 (2.15–5.20)	<0.001		2.17 (1.38–3.41)	0.001
North Africa and Middle East	0.77 (0.59–1.00)	0.047		1.01 (0.75–1.38)	0.928
Southeast Asia	3.35 (2.72–4.12)	<0.001		2.65 (2.15–3.28)	<0.001
Southern and Central Asia	1.19 (0.96–1.47)	0.120		0.84 (0.67–1.05)	0.124
Americas	0.96 (0.48–1.90)	0.900		0.79 (0.40–1.57)	0.498
Sub-Saharan Africa	1.28 (0.96–1.69)	0.088		0.84 (0.63–1.12)	0.224
Visa stream					
Permanent	Referent	<0.001		Referent	<0.001
Humanitarian	0.59 (0.51–0.70)			0.47 (0.38–0.58)	
Risk factors					
Past close contact with TB	3.48 (2.15–5.61)	<0.001		2.80 (1.70–4.61)	<0.001
Test type					
IGRA	Referent	<0.001		Referent	<0.001
TST	2.72 (2.41–3.06)			3.01 (2.66–3.42)	

*Indeterminate IGRA results excluded. Temporary applicants excluded as not included in screening unless clinical features or risk factors. Applicants with TB and history of TB excluded, because likely to result in positive IGRA or TST without reflecting LTBI. Screened applicants without country of origin data not included in the regression analysis (n = 2,383); the LTBI prevalence in this group was 2.5% (Table 2). IGRA, interferon-gamma release assay; LTBI, latent tuberculosis infection; TB, tuberculosis disease; TST, tuberculin skin test.

### Further Investigation and Linkage to Care

Almost all applicants (1,495/1,513 [98.8%]) with positive IGRA or TST completed posteroanterior chest radiography; a lateral film was also performed in 97.1% (1,469/1,513) applicants. Only 2.7% (41/1,495) of radiographs demonstrated any findings consistent with new or old TB; 10 of those children received a TB diagnosis.

During November 2015–June 2017, a total of 21 cases of TB were diagnosed among 134,759 children, of which 1 was bacteriologically confirmed, for a prevalence of 15.6 cases/100,000 child applicants. Of these 21 TB-positive children, 4 did not undergo IGRA or TST, 10 had positive results (8 TST, 2 IGRA), and 7 had negative results (all IGRA). All TB cases had clinical abnormalities, radiological abnormalities, or both on IME.

During November 2015–June 2017, among offshore applicants, 1,640 children were allocated TBHUs; the greatest number was in those 5–9 years of age (792/1,640 [48.3%]; p<0.001) and permanent applicants (1,113/1,640 [67.9%]; p<0.001). TBHUs increased significantly after new screening introduction. Comparing the time periods July 2014–October 2015 and November 2015–June 2017, TBHUs increased from 417 (279/66,887) to 1,751 (1,640/93,650) per 100,000 offshore child applicants (p<0.001). Among offshore applicants with positive IGRA or TST results, 1,245/1,327 (93.8%) were allocated TBHUs. Of note, 183 onshore applicants had positive IGRA or TST results and were likely referred to care in Australia. Thus, 1,428/1,513 (94.4%) of all applicants with positive IGRA or TST results were likely referred for further care.

## Discussion

Given the primary aim of the new screening program, we found a low yield in detecting TB. TB is rare in children migrating to Australia; 15.6 cases per 100,000 child applicants were diagnosed over the study period, and annual Australia TB notifications in overseas-born children ranged from 4.5 to 8.8 cases per 100,000 children during 2015–2018 (*7*). Further, the effect of including IGRA and TST in screening on case detection is uncertain. Of the 17 TB-positive children in whom IGRA or TST was performed, 7 had a negative test for infection (all IGRA), and in the other 10 children, it cannot be assumed that the positive IGRA or TST result led to the diagnosis of TB. Although a test for infection is often included in the approach to TB diagnosis in children, the results do not confirm or exclude active disease and so cannot be interpreted in isolation (*25*,*26*).

IGRA and TST detect LTBI in children, who might benefit from TB preventive treatment (*4*). We found that 3.3% of children screened had LTBI, equating to 30 applicants screened per LTBI case detected. Factors associated with LTBI matched well-established risk factors, namely increasing age, history of TB contact, and coming from a region with high TB prevalence (acknowledging variability within regions). These findings support previous evidence showing greater yield from migrant TB screening targeting higher-risk groups (*14*). Increased positivity with TST versus IGRA also reflects known test characteristics; IGRA has greater specificity in BCG-vaccinated children (*26*).

Although the LTBI prevalence of 3.3% found in this population was low, that prevalence was higher than the recently modeled estimate of 2.1% for overseas-born children (0–14 years of age) in Australia (*12*), likely reflecting targeted screening criteria. LTBI prevalence might have been higher if children 11–14 years of age had also been screened, given LTBI prevalence increases with age in TB-endemic settings. LTBI prevalence was much lower than in some other international migrant screening programs. An analysis of premigration LTBI screening in 67,334 children 2–14 years of age bound for the United States (from countries with TB incidence rate >20 cases per 100,000 children) found a higher LTBI prevalence of 12% (*27*).

Characteristics of those screened largely reflected screening criteria. Although overall screening completeness could not be determined, screening was nearly universal among humanitarian applicants 5–9 years of age, who all met criteria. Although complete assessment of the risk factors prompting testing in temporary applicants was not possible, a surprisingly high proportion (9.8%) of temporary applicants underwent IGRA or TST; the proportion testing positive (3.6%) was similar to the proportion in permanent applicants (3.8%). In contrast, humanitarian applicants had significantly lower odds of LTBI than permanent applicants despite the presumption of high LTBI risk and mandated testing. This difference is likely because permanent applicants were only screened if they were from a high-burden setting, and the humanitarian intake at the time was predominantly from countries with relatively low TB prevalence (largely refugee applicants from Syria and Iraq) (*28*). Nevertheless, the proportion of positivity (2.1%) in humanitarian applicants was lower than previous LTBI prevalence estimates in refugee-background children tested in Australia, including from the Middle East (*11*,*29*).

Almost all children with positive screening results had further investigation for TB, including chest radiography, as per guidelines. Most were allocated a TBHU requiring linkage to follow-up care in Australia. The updated screening was associated with a marked increase in children with TBHUs. These additional TBHUs might have enabled TB preventive treatment that might not otherwise have occurred, for potential substantial benefit for these children. However, TBHUs require use of services and resources both for families and the health system in Australia. Time from visa application to follow up after migration is often months to years. This delay from a positive test to commencement of TB preventive treatment (in addition to the unknown time between infection and positive test) risks missing the period when disease is more likely to develop in children with LTBI (*5*). Which might reduce both the individual and public health benefit of preventive treatment.

After migration, Australia guidelines recommend testing migrants <35 years of age from high-burden TB settings for LTBI (*30*). In practice, this testing occurs infrequently unless migrants are assigned a TBHU, linked with specific services (e.g., refugee health clinics), or there is another specific requirement (e.g., occupational screening) (*31*,*32*). Systematic premigration LTBI testing linked to appropriate follow-up care theoretically offers several advantages, including broader testing within mandated IMEs, targeting migrants from high-risk settings, supporting source country TB infrastructure, and detecting infection promptly, given LTBI reactivation risk is highest early after migration (*21*,*33*–*35*). Some migrant groups have also reported a preference for premigration LTBI testing over postmigration testing (*36*). Researchers analyzing premigration TB screening (with chest radiography) suggest considering expansion to include LTBI, citing reactivation of overseas-acquired LTBI as the primary driver of TB in low-burden countries (*13*,*33*,*37*,*38*). Some reviews and modeling estimates have supported the potential effectiveness and cost-effectiveness of premigration LTBI screening, particularly when targeted to migrants from high-burden settings (*16*,*33*). Others have found limited cost-effectiveness, particularly if screening costs are included in cost-effectiveness calculations (rather than being borne by migrants), and have highlighted the limited likely contribution of migrant screening to TB elimination in low-burden settings (*39*). However, observational data from premigration health screening programs detecting LTBI, particularly in children, remain limited, underscoring the importance of analyses such as this one (*33*,*40*).

Ideally, assessment of the overall effectiveness of the new screening program would consider the full cascade of care, from premigration LTBI detection to postmigration TB preventive treatment uptake and completion, as well as TB incidence (*33*). Losses over the cascade of care are common: of 8,231 children in whom LTBI was detected before migration to the United States, 70% were followed after migration but rates of diagnosis revision and low treatment completion were substantial (*27*). However, given that TB will not develop in most children with LTBI even without TB preventive treatment, the low LTBI prevalence in our analysis implies a large number of children would need to be screened to prevent 1 case of TB.

The first limitation of our analysis is that the study population represents visa applications rather than persons, meaning repeat applicants are not accounted for (*22*). No data were available regarding BCG immunization. Of note, BCG vaccine is included in the immunization schedule of many applicant source countries (e.g., India, China, and the Philippines), and a large proportion of screened applicants likely received the BCG vaccine in infancy. However, in TB-endemic countries, a positive TST indicates infection with *M. tuberculosis* regardless of BCG status, and false positivity decreases with increasing age (*41*,*42*). Further, in children, a positive TST requires clinical follow up regardless of BCG status.

We were unable to assess screening completeness directly against criteria because of privacy limitations on data format. Our denominator for the proportion of children screened was inflated by including children <2 years and 11–14 years of age, whose age placed them outside of screening criteria. Because of these factors, the cohort represents a mix of children screened based on age, visa stream, and clinical risk criteria. Nevertheless, the demographics of the cohort broadly align with screening criteria, and sensitivity analyses restricted to children 5–9 years of age did not alter our conclusions, suggesting our group largely reflects children meeting screening criteria. Further, our results reflect real-world implementation of the updated screening program. Finally, no data were available regarding care linkage in Australia or clinical outcomes.

Our analysis provides the results of Australia’s systematic premigration TB screening in children, one of few such programs globally. Notwithstanding inherent delays between infection, screening, and TB preventive treatment, the individual benefit for children with LTBI detected by premigration health screening could be considerable, with potential future reductions in TB incidence in overseas-born children in Australia. However, the low proportion of LTBI found in our analysis, despite the program targeting higher-risk children, suggests a modest yield from the current program toward substantial reductions in TB after migration.

TB elimination in Australia is unlikely to be achieved through migrant screening alone, and strategies such as investment in TB programs in high-burden settings, alongside strong local public health measures, might have greater impact (*10*,*39*,*40*). Key questions remain unanswered that will influence whether, and how, migrant screening can best contribute to TB and LTBI detection. These questions include the effects of changing migration patterns on screening yield, trade-offs between screening older migrants (with higher infection prevalence) versus children (with greater individual benefit for those with LTBI), acceptability and cost for migrants, health resourcing implications, and ultimately, effectiveness for reducing TB burden, toward the overarching goal of TB elimination.

In conclusion, in low-burden settings, additional interventions to prevent TB are inherently likely to have lower yield and be less cost-effective because of already low TB incidence. Shifting screening costs onto migrants might improve apparent cost-effectiveness but has implications for equitable access to screening. Given TB epidemiology in Australia, targeted migrant screening might still make a valuable contribution toward TB elimination even if absolute yield is low.
